# Activation-dependent learning rule for GPCR localization - 5ht2AR regulation in prefrontal cortical neurons

**DOI:** 10.1186/1471-2202-14-S1-P344

**Published:** 2013-07-08

**Authors:** Gabriele Scheler

**Affiliations:** 1 Carl-Correns-Foundation, Mountain View, CA 94040, USA

## 

Serotonin (5-ht) through 5ht2A receptors increases neuronal excitability (the neuronal gain function) by blocking voltage-gated K+ channels (Kv1.5) [1]. 5ht2A receptors also reduce calcium influx and activate calcium from intracellular stores (Ca[i]) [2]. High agonist exposure generally decreases 5ht2A density through endocytosis [3], in contrast 5ht2A receptors are upregulated after 5-ht innervation loss or under low 5ht exposure [4]. Both adaptations usually requires increase of Ca[i] and/or PKC pathway activation. We want to understand the contribution of serotonin receptor and other GPCR regulation on neuronal plasticity by the differential expression level of these receptors and the conditions for their regulation. As a hypothesis to guide further research, we propose to use a ***learning rule ***for 5ht2A placement, such that receptor density (R) is decreased by high agonist, or increased by low agonist, but only in the presence of high internal calcium.

$$\Delta \text{R}\text{ = }\text{X}\left(\left[5\text{ht}\right]-\left[5\text{ht}\right]\text{m}\right)\gamma \left(\left[\text{Cai}\right]/\left[\text{Cai}\right]\text{m}\right)$$

Here [5ht]m is the average 5ht concentration, [Cai]m the average calcium concentration, 5ht is the actual concentration, x and γ are scaling factors. We further assume that [Cai] reflects the history of neuronal activation.

This learning rule may now be used in a network of model neurons. Principal neurons in prefrontal cortex (PFC) are initialized with a random distribution of 5ht2A receptors linked to voltage-gated K+ channels to regulate neuronal excitability. The model contains glial cells as variable sinks or sources of glutamate, to stabilize network activation, and a set of interneurons which contain activating 5ht2a receptors [5]. Synaptic connectivity is initially random.

The model is loaded with patterns which produce a characteristic recurrent neural representation, determined by the synaptic connectivity and the 5ht2A-regulated intrinsic gain functions [1]. The model is trained under 5ht stimulation of varying magnitudes. 5ht increases excitability of neurons in a dose-dependent manner, and the density of 5ht2A receptors affects the intrinsic gain functions. We can show that patterns that were learned under high serotonin contain neurons with decreased 5ht sensitivity. When similar patterns are re-eoncountered under average or low serotonin conditions, these (the overlapping) features will be suppressed. Since the relevant neurons have decreased response to serotonin, they also have decreased excitability. The model could be altered to use dendritic regions rather than neurons. Since 5ht memory affects neuronal excitability, we have intrinsically generated differences in firing rates that start to affect synaptic connectivity. This creates traces in synaptic connectivity independent of 5ht activation. If 5ht2AR memory is erased, the trace is still present. Synaptic connectivity also determines which neurons (dendritic regions) receive sufficient input to undergo 5ht-mediated plasticity. The transfer of memory between synaptic connectivity and G-protein coupled receptor (GPCR) localization is therefore a two-way process. Memory may be stored in different places at different times. The model is undoubtedly simplistic, since many co-regulations among GPCRs exist, which are cell-type specific and require detailed analysis. But it directly explains findings such as [6], where 5-HT2AR stimulation produces an increase in activity for preferred target locations and/or a reduction in activity for nonpreferred locations in a working memory task in PFC. Most importantly, it provides a unified starting point to understand the regulation of GPCR distribution as a method for pattern storage that is synergistic with but not identical to AMPA synaptic strengthening or weakening, and may play an as yet not well understood role in the maintenance of a synapse.
